# DNA methylation dynamics during *ex vivo* differentiation and maturation of human dendritic cells

**DOI:** 10.1186/1756-8935-7-21

**Published:** 2014-08-20

**Authors:** Xue Zhang, Ashley Ulm, Hari K Somineni, Sunghee Oh, Matthew T Weirauch, Hong-Xuan Zhang, Xiaoting Chen, Maria A Lehn, Edith M Janssen, Hong Ji

**Affiliations:** 1 Division of Human Genetics, Cincinnati Children’s Hospital Medical Center, 3333 Burnet Ave, Cincinnati, OH 45229, USA; 2 Division of Asthma Research, Cincinnati Children’s Hospital Medical Center, 3333 Burnet Ave, Cincinnati, OH 45229, USA; 3 Division of Human Genetics, Kim Sook Za Children’s Hospital Medical Center Research Foundation, 745 JikJi Daero Heung Deok Gu, Cheongju, Chung Buk 361-841, South Korea; 4 Center for Autoimmune Genomics and Etiology and Division of Biomedical Informatics, Cincinnati Children’s Hospital Medical Center, 3333 Burnet Ave, Cincinnati, OH 45229, USA; 5 Procter & Gamble Co., Mason Business Center, 8700 S Mason Montgomery Road, Mason, OH 45040, USA; 6 School of Electronic and Computing Systems, University of Cincinnati, Cincinnati, Ohio 45221, USA; 7 Division of Immunobiology, Cincinnati Children’s Hospital Medical Center, 3333 Burnet Ave, Cincinnati, OH 45229, USA

**Keywords:** DNA methylation, Human dendritic cells, Monocytes, Differentiation, Maturation, TET, DNMT

## Abstract

**Background:**

Dendritic cells (DCs) are important mediators of innate and adaptive immune responses, but the gene networks governing their lineage differentiation and maturation are poorly understood. To gain insight into the mechanisms that promote human DC differentiation and contribute to the acquisition of their functional phenotypes, we performed genome-wide base-resolution mapping of 5-methylcytosine in purified monocytes and in monocyte-derived immature and mature DCs.

**Results:**

DC development and maturation were associated with a great loss of DNA methylation across many regions, most of which occurs at predicted enhancers and binding sites for known transcription factors affiliated with DC lineage specification and response to immune stimuli. In addition, we discovered novel genes that may contribute to DC differentiation and maturation. Interestingly, many genes close to demethylated CG sites were upregulated in expression. We observed dynamic changes in the expression of *TET2*, *DNMT1*, *DNMT3A* and *DNMT3B* coupled with temporal locus-specific demethylation, providing possible mechanisms accounting for the dramatic loss in DNA methylation.

**Conclusions:**

Our study is the first to map DNA methylation changes during human DC differentiation and maturation in purified cell populations and will greatly enhance the understanding of DC development and maturation and aid in the development of more efficacious DC-based therapeutic strategies.

## Background

Dendritic cells (DCs) are a heterogeneous group of bone marrow-derived cells within various organs, which display different cell surface phenotypes and serve different functions depending on location, development, and activation status. DCs bridge two arms of the immune response: the innate immune response via the recognition of pathogens through pattern-recognition receptors and the adaptive immune response via the activation of T and B cells [1]. They can exist in two developmental states, immature (iDC) and mature (mDC), with alternate functional characteristics in each state. The induction of DC differentiation *ex vivo* from human and mouse peripheral monocytes by granulocyte-macrophage colony stimulating factor (GM-CSF) and Interleukin 4 (IL4) suggest that monocytes may serve as an important reservoir for DC development [2]. Mouse studies also support that monocytes can develop *in vivo* into a DC-like population [3]. Like conventional DCs (cDCs), GM-CSF and IL-4 derived DCs (iDCs) upregulate their expression of CD11c and major histocompatibility complex (MHC) class II complexes and efficiently stimulate naive T cells [4]. A widely accepted cytokine mix can further transform iDCs into mDCs [5]. With the FDA approval of the antigen-presenting cell vaccine sipuleucel-T for prostate cancer, DC-based therapeutic vaccines have become an established approach for the treatment of established cancer. In human blood, two major phenotypically and functionally distinct DC populations have been described, the CD11c^+^ CD123^-^ myeloid DCs and the CD11c^-^ CD123^+^ plasmatoid DCs. The myeloid DCs have been further defined into three subsets based on the expression of CD16, BDCA-1 and BDCA-3 [6]. Recently, it has been demonstrated that human BDCA3^+^ DCs possess characteristics of mouse CD8α^+^ DCs and can induce cytotoxic T lymphocyte responses [7,8], and therefore, are the most relevant targets for vaccination against cancer. Due to the complexity of the lineage and difficulty in lineage determination based on surface markers, the molecular mechanisms regulating the development of DCs are not well understood compared to other lineages such as T cells [9]. Studying the *ex vivo* differentiation of monocytes into DCs may help us better understand the differentiation of different DC subtypes *in vivo* and allow for the successful generation of more efficacious DC vaccines in the future.

As an epigenetic mechanism that regulates gene expression both *in cis* and *in trans*, DNA methylation has been shown to regulate gene expression of related pathways and cellular identity in the immune system [10-13]. In mammalian cells, DNA methylation is maintained by DNA methyl-transferases DNMT1, DNMT3A and 3B. DNMT1 methylates hemi-methylated parent-daughter duplexes during DNA replication, while *de novo* methylation is predominantly carried out by DNMT3A and 3B. Several promising, yet controversial, mechanisms have been proposed for DNA demethylation, such as the deamination of 5mC to T, coupled with G/T mismatch repair by DNA glycosylases [14], or the hydroxylation of TET proteins through the generation of 5-hydroxymethylcytosine (5hmC) and 5-formylcytosine (5fC) [15-17]. The combination of methylation by DNMTs and demethylation by TETs may contribute to the observed dynamic DNA methylation changes during cellular differentiation [10]. DNA methylation is a potential mechanism governing the differentiation and activation of DCs. Indeed, locus and region-specific DNA methylation changes have been observed during the *ex vivo* differentiation of monocytes to iDCs [12,18]. A detailed study of DNA methylation dynamics during these processes will greatly help to better tease apart the molecular events that occur during the transition from monocytes to iDCs, and from iDCs to mDCs.

In this study, we established genomic maps of DNA methylation at single nucleotide-resolution for human monocytes and monocyte-derived immature and mature DCs [19]. Besides identification of genes and pathways known to be involved in DC differentiation and maturation, we observed dynamic DNA methylation changes at many novel genes, most of which are demethylated. Interestingly, these changes occur close to the binding sites of transcription factors that are implicated in DC differentiation and function. In addition, we correlated DNA methylation levels at differentially methylated sites/points (DMPs) with expression levels of genes located within 1,500 bp distance using published gene expression arrays and found a general inverse correlation between DNA methylation and gene expression levels. Time course experiments showed that the demethylation event is locus-specific, and is coupled with dynamic changes in the DNA methylation machinery, including TET2, DNMT1, DNMT3A and DNMT3B. Besides providing detailed DNA methylome reference maps for purified monocytes, iDCs and mDCs, our study demonstrated the dynamic epigenetic regulation of genes and pathways important for DC development and maturation, which are potential targets to improve DC-based therapeutic strategies.

## Results

### Genome-wide scanning identifies DNA methylation changes during dendritic cell differentiation and maturation

**Figure 1:**
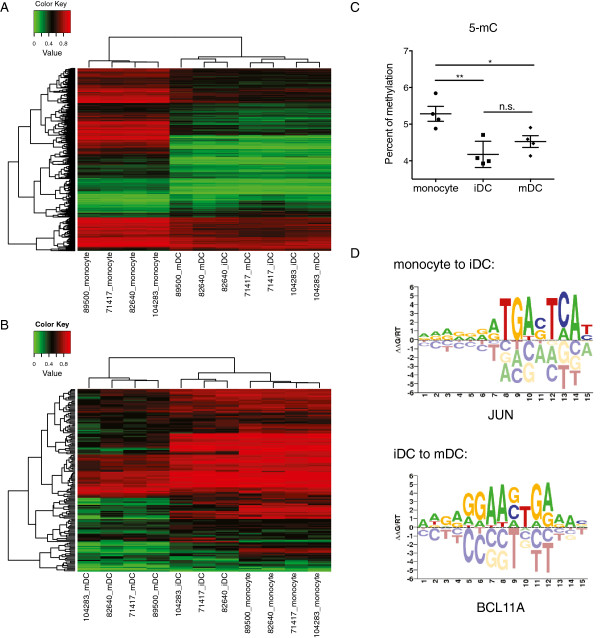
**DNA methylation changes occur at non-CGI and transcription factor binding sites during dendritic cell (DC) differentiation and maturation. A)** Heatmap showing the CG sites with greater than 10% methylation difference between monocytes and immature dendritic cells (iDCs) among all samples. **B)** Heatmap showing the CG sites with greater than 10% methylation difference between iDCs and mature dendritic cells (mDCs) among all samples. **C)** Global DNA methylation levels measured by 5mC ELISA in monocytes, sorted iDCs and sorted mDCs used for microarray analysis. Four technical replicates were used in each cell type, and results are shown as mean ± SD. *P <0.05, **P <0.01, n.s., nonsignificant, paired t test. **D)** Examples of potential transcription factor binding sites within 50 bp around demethylated CG sites.

We *ex vivo* differentiated monocytes (from four blood donors) into iDCs and matured them using the Jonuleit cytokine cocktail mix (IL-1β, IL-1α, IL-6, TNF-α and PGE_2_) following the established FDA approved protocol [see Additional file 1A] [5]. The iDCs (HLA-DR^low^) and mDCs (CD83^+^, CD86^+^ and HLA-DR^high^) were fluorescence-activated cell sorting (FACS) purified (>95% purity) and subjected to further analysis [see Additional file 1B]. Using a cutoff of *P* value ≤0.05 and absolute difference ≥0.1, we identified 1,608 DMPs from monocytes to iDCs and 156 DMPs from iDC to mDCs (Table 1, Figure 1A and B). Only 6% of the identified DMPs are located within CpG islands even though 31% of CG sites assayed are within CpG islands. Consistent with previous observations, our findings support that the DNA methylation level of CGs at shores and shelves (defined as regions that are 0 to 2 kb and 2 to 4 kb away from CpG islands, respectively) may be more dynamic and critical during cellular differentiation than that of CpG islands [10]. Interestingly, the vast majority of these sites are demethylated (1,367 out of 1,608 DMPs from monocytes to iDC and 139 out of 156 DMPs from iDC to mDC) (Figure 1 and Table 1). We further measured whole-genome DNA methylation levels using an ELISA-based method, and confirmed the occurrence of CG demethylation during iDC differentiation from monocytes (Figure 1C). A total of 933 genes are linked to the 1,608 DMPs with DNA methylation changes from monocytes to iDCs (Table 1). Among these genes, 795 genes are exclusively linked to demethylated DMPs, 117 genes are linked to more methylated DMPs, and 21 genes (2%) are linked to DMPs with methylation changes in both directions [see Additional file 2A, B and C]. A total of 116 genes are linked to the 156 DMPs from iDC to mDCs, among which 102 genes are linked to DMPs, all with reduced methylation, and the rest of 14 genes are linked to DMPs, all with increased methylation [see Additional file 2D and E].

**Table 1:** **Numbers of differentially methylated CG sites (DMPs) identified during *ex vivo *dendritic differentiation and maturation**

**Comparisons (G1 versus G2)**	**Numbers of DMPs**^**a**^		**Locations of DMPs relative to CpG islands (%)**				**Enhancer associated**^**e**^	**Promoter associated**^**f**^	**Gene associated**^**g**^
	**G1 > G2**	**G1 < G2**	**Islands**	**Shores**^**b**^	**Shelf**^**c**^	**>4 kb**^**d**^			
CD14^+^ versus immature dendritic cell (iDC)	1368	240	5.9	23.5	16.1	54.5	701	139	933
iDC versus mature dendritic cell (mDC)	139	17	3.3	18.2	14.9	63.6	80	9	116

^a^*P* value cutoff of 0.05, difference change of 10% in DNA methylation was used to calculate the number of DMPs (see Methods).

^b^Shores were defined for this table as 2 kb from an island (N and S island).

^c^Shelves were defined for this table as 2 kb-4 kb away from an island (N and S shore).

^d^Regions over 4 kb from an island.

^e, f, g^Based on HumanMethylation450 v1.2 Manifest File (http://support.illumina.com/downloads/infinium_humanmethylation450_product_files.html).

A great number of the identified demethylated CG sites are located at enhancers (Table 1, 43.6% CG sites from monocyte to iDCs and 51.6% from iDC to mDC). Previous studies have shown that chromatin markers including H3K4me1, H3K4me3 and H3K27Ac are enriched at human enhancers [20,21] and are highly cell-type specific. We examined the overlap between H3K4me1, H3K4me3 and H3K27Ac markers in monocytes [22] with CG sites that undergo DNA methylation changes from monocyte to iDCs, and found that 67.6% of the CG sites have H3K4me1 markers (45.5% CG sites from iDC to mDC). More than half of these CG sites with H3K4me1 also have H3K4me3 and H3K27Ac markers.

We then searched for transcription factor binding sites in 51 bp windows centered on these DMPs and identified several transcription factors with known roles in dendritic cell lineage specification (Figure 1D, Additional file 3, see Methods). The consensus sequence of the most strongly enriched motif for the monocyte to iDC transition is TGACTGA, the AP-1 response element bound by bZIP transcription factors JUN, FOS, BATF, BATF3, as well as IRF4, and IRF8 [23]. Among these, IRF8 is a transcription factor that distinguishes a DC-committed progenitor from myeloid progenitors [24] and is important for the development of several DC subsets [25,26]. In addition, BATF3 binds to JUN and is required for the normal development of CD8α^+^ cDCs in mouse models and BDCA3^+^ DCs in humans [27-29]. Furthermore, IRF4 interacts with PU.1 and is required for the development of CD11b^+^ cDCs [30]. Motifs most strongly enriched for the iDC to mDC transition contain a GGAA core, which binds to transcription factors including BCL11A, SPIB and RELA. Indeed, BCLLA and SPIB may regulate pDC development [31,32], while RELA is a NF-κB family member that regulates CD11c^+^ DC generation [33] and cytokine production in myeloid DCs [34].

### Pathway analysis reveals significant genes and networks during dendritic cell differentiation

We next performed pathway analysis to identify biological processes that undergo DNA methylation changes during DC differentiation and maturation, stratified by directions of change [see Additional files 4 and 5]. First, components of the IL-4 and GM-CSF signaling pathways were demethylated, consistent with our approaches and suggesting that these molecules induce DC differentiation from monocytes through the modification of DNA methylation. The genes involved in cytokine production and interaction with T cells, such as IL-6, IL-10, IL-12 and T cell receptor signaling were demethylated when exposed to differentiation stimuli [see Additional file 4A], indicating an epigenetic priming of iDCs by IL-4 and GM-CSF for their secretion of cytokines and activation of naïve T cells.. Consistently, upstream regulator analysis in Ingenuity Pathway Analysis (IPA) revealed that targets of IL-1α, IL-1β, IL-6 and TNF-α are differentially methylated in iDCs by IL-4 and GM-CSF induction [see Additional file 6]. These cytokines are included in the DC maturation cocktail, supporting a priming process for the response of the immature DCs to cytokine stimuli.

**Figure 2:**
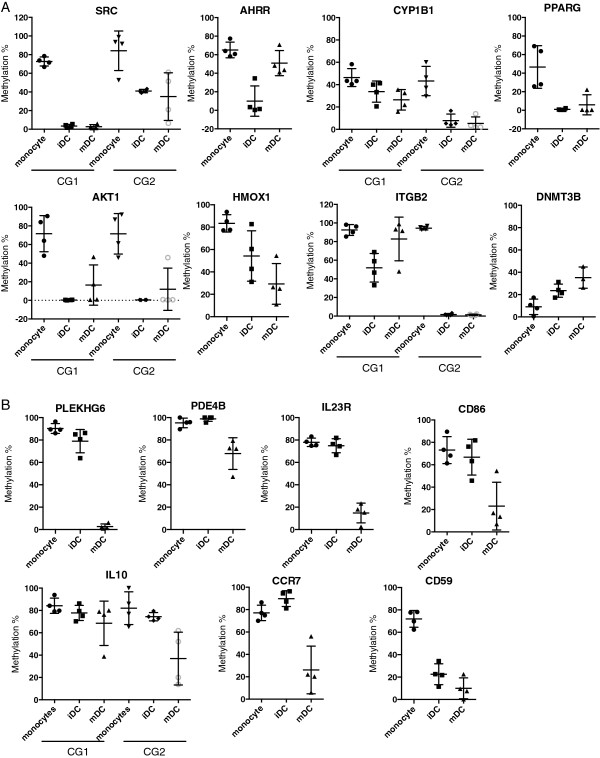
**Locus-specific bisulfite sequencing validates microarray findings.** DNA methylation changes in *SRC*, *AHRR*, *CYP1B1*, *PPARG*, *AKT1*, *HMOX1*, *ITGB2*, and *DNMT3B***(A)**, and *PLEKHG6*, *PDE4B*, *IL23R*, *CD86*, *IL10*, *CCR7* and *CD59***(B)**. Four technical replicates of flow-purified cells were used in each cell type, and results are shown as mean ± SD.

Second, an IPA search on demethylated genes from monocytes to iDCs [see Additional file 2A] resulted in a number of enriched pathways including Aryl Hydrocarbon Receptor signaling (AhR), PPAR signaling, AKT signaling, Integrin signaling, IL-6 signaling, IL-10 signaling, IL-12 signaling and production, T cell receptor signaling, NRF2-mediated oxidative stress response, granulocyte adhesion and diapedesis, caveolae-mediated endocytosis signaling, clathrin-mediated endocytosis signaling, and macropinocytosis signaling (Figure 2A and Additional file 4A). Interestingly, many of these demethylated pathways are required for DCs to recognize and process antigens and present them to T cells, which is in line with the functional characteristics of iDCs (Figure 2A). For example, *SRC* (Rous sarcoma oncogene) encodes a tyrosine-protein kinase that participates in many immune pathways. Proteins encoded by *SRC*, *AHRR* (aryl-hydrocarbon receptor repressor), and *CYP1B1* (cytochrome P450, family 1, subfamily B, polypeptide 1) participate in the AhR signaling cascade, which mediates the response of DCs to dioxin [35-37], and is involved in the regulation of normal immune cell development and the immune response of DCs following lipopolysaccharide challenge or influenza virus infection [38,39]. *PPARG* (peroxisome proliferator-activated receptor gamma) encodes a lipid-activated transcription factor that positively regulates myeloid DC maturation and functions [40-43]. However, the detailed roles of each of the genes in DC differentiation and maturation are unclear. We also observed an 80% decrease in DNA methylation at a CG site located within the second intron of *AKT1* (v-akt murine thymoma viral oncogene homolog 1). AKT plays a critical role in proinflammatory-mediated DC survival and maturation; human monocyte-derived DCs with constitutively active lipid raft-targeted AKT1 survived significantly longer and had promoted antigen-specific T-cell responses, while downregulation of *AKT1* reduced their life span [44]. In addition, the methylation level at a CG site located at the 3′ UTR of *HMOX1* (heme oxygenase 1) was found to be reduced. The heme oxygenase 1 (HO-1) encoded by this gene is a stress responsive gene whose expression is induced by a variety of stimuli including heme, heavy metals, inflammatory cytokines, and nitric oxide (reviewed in [45]). Induction of HO-1 inhibits lipopolysaccharide (LPS)-induced dendritic cell phenotypic maturation and the secretion of proinflammatory cytokines, resulting in the inhibition of alloreactive T-cell proliferation [46,47]. The activation of these genes is consistent with the role of iDC in taking up antigens by phagocytosis or macropinocytosis and processing the internalized antigen.

### Pathway analysis reveals significant genes and networks during dendritic cell maturation

From iDC to mDC, the pathways involved in communication between innate and adaptive immune cells, the complement system, and cross talk between DCs and natural killer cells were demethylated [see Additional file 5A]. This is consistent with the function of mDC in antigen presentation to effector cells through the expression of MHC and membrane-associated co-stimulatory molecules, and secretion of co-stimulatory cytokines. Specifically, we found that CG sites located in myosin-interacting guanine nucleotide exchange factor (*PLEKHG6*), phosphodiesterase 4B (*PDE4B*), interleukin 23 receptor (*IL23R*), *CD86*, interleukin 10 (*IL10*) and chemokine receptor 7 (*CCR7*), and *CD59* were significantly demethylated from iDC to mDC (Figure 2B). Among these genes, the expression of *PDE4B* is low in iDCs and is upregulated in mDCs (GSE7509) [48], which is consistent with its high methylation in iDCs and reduced DNA methylation level in mDCs. Addition of PDE4 inhibitor during DC maturation impairs IL12 and TNF-α production in response to LPS and CD40 ligand and destroys the capacity to generate Th1 cells [49]. *IL23R* encodes interleukin-23 receptor, which pairs with IL12RB1 to form the receptor for IL-23A/IL-23. This receptor is expressed in T cells, and IL-23A signaling is required for the survival and/or expansion of Th17 cells [50]. Although this protein is also expressed on monocyte-derived DCs (GSE6965) [51], the role of IL23R in DC maturation and function is unknown. Further, CD86 is a surface marker for DCs, and binding of this protein to CD28 acts as a co-stimulatory signal for T cell activation by DCs [52,53]. The methylation at a CG site located in the *IL10* promoter is reduced in mDCs compared to iDCs. IL10 may regulate DC differentiation and maturation because addition of IL10 to the differentiation cocktail generates mDCs with distinct phenotypes [54] and these DCs have an impaired capacity to induce a Th1-type response *in vivo*, leading to the development of Th2 lymphocytes [55]. CCR7 is a chemokine receptor necessary to direct dendritic cells (DCs) to secondary lymphoid nodes and to elicit an adaptive immune response [56-58]. Similarly, as a cell surface glycoprotein that regulates complement-mediated cell lysis, CD59 is constitutively expressed in monocyte derived-DCs. However, how this protein regulates DC function is not clear. Maturation by LPS or TNF-α/IL-1/PGE_2_ significantly increases CD59 expression [59], probably through demethylation of the promoter of *CD59* as suggested by our study.

### DNA methylation changes are correlated with gene expression profiles

**Figure 3:**
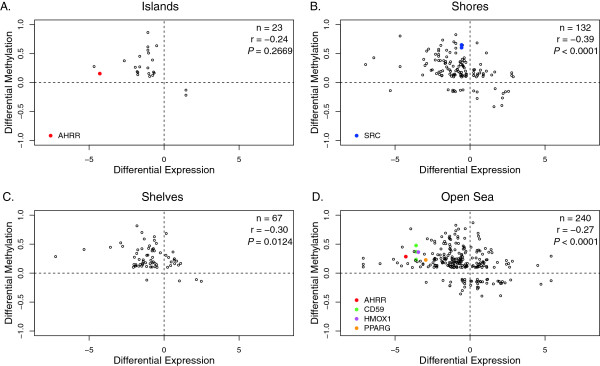
**Correlation of DNA methylation changes with gene expression alterations during dendritic cell (DC) differentiation.** CG sites with significant DNA methylation changes were grouped by its relative distance to CGI. **A**. CpG island; **B**. CG shores: 0 to 2 kb from an island; **C**. Shelves: 2 to 4 kb from an island; **D**. Open sea: 4 kb away from an island. The changes in expression levels of differentially expressed genes (false discovery rate (FDR) <0.05) were then correlated with changes in beta values of differentially methylated points (DMPs), and Pearson’s correlation coefficients were reported for CG sites in each category. CG sites validated in Figure 2 whose associated gene expression levels also significantly altered were highlighted with different colors.

DNA methylation provides a mechanism for robust and epigenetically heritable gene silencing. DNA methylation at CpG islands has been the focus of many studies and is generally associated with gene silencing. However, significant methylation changes do not occur exclusively at CpG islands. CpG island shores and shelves are also closely associated with transcriptional silencing [10,60,61]. To further understand the role of observed DNA methylation changes, we correlated our DNA methylation data with publically available gene expression data (Affymetrix Human genome U133 Plus 2) for monocytes, iDCs and mDCs (GEO: GSE7509) [48,62]. In general, we found a significant negative correlation between DNA methylation and gene expression at differentially methylated loci from monocytes to iDCs at CpG shores, shelves and open sea (Figure 3). This is in agreement with previous findings in hematopoietic lineage commitment, and highlights the significance of DNA methylation in gene silencing. Several validated demethylated CG sites are associated with a significant increase in gene expression (Figure 3). From iDCs to mDCs, there is a similar relationship between DNA methylation and gene expression, although none of them reached statistical significance likely due to the smaller number in each category [see Additional file 7].

### Expression of DNA methylation machinery couples with dynamic DNA methylation changes

**Figure 4:**
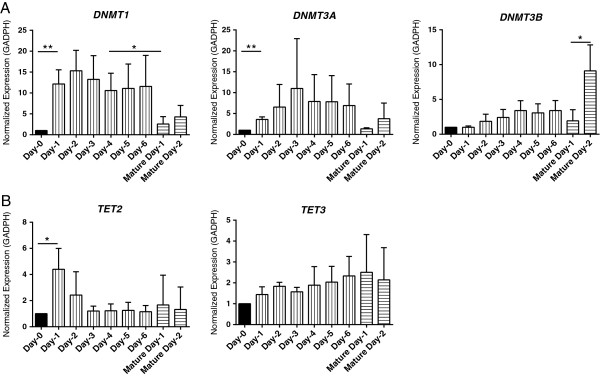
**Dynamic changes in expression levels of *TET2/3 *and *DNMTs*.** Gene expression levels of *DNMT1*, *DNMT3A*, and *DNMT3B***(A)**, *TET2*, *TET3* in **(B)**. Independent experiments for each donor were performed and expression levels were normalized to GADPH, and three biological replicates were used in each condition. Bar graphs shown in this figure represent pooled data analyses from all the four independent experiments. Comparisons were made for Day-0 versus Day-1, Day-4 versus Mature Day-1, and Mature Day-1 versus Mature Day-2 using paired t-test. *P <0.05, **P <0.01.

Among the DMPs we discovered, we have observed extensive demethylation during monocyte differentiation into iDCs and from iDCs to mDCs (Table 1, Figure 1A and B). One of the mechanisms that could account for this demethylation is the downregulation of DNA methyl-transferases, including DNMT1, DNMT3A and DNMT3B. Surprisingly, independent analyses of monocytes, iDCs and mDCs generated from individual donors showed a significant increase in the expression levels of *DNMT1* and *DNMT3A* during the differentiation from monocyte to iDC (day 1 to 4 versus day 0) and a significant decrease at 24 hours after the addition of maturation cocktail (mature day-1 versus day-4) [see Additional file 8A]. However, the later significance with *DNMT3A* was lost when we analyzed pooled data from four individual donors, possibly due to the variability between donors (Figure 4A). The respective increase and decrease in the expression levels of *DNMT1* during differentiation and maturation was in agreement with the previously published dataset (GEO: GSE7509). However, our finding of *DNMT3A* during the differentiation of monocyte to iDC contradicts the public dataset, where they reported a significant decrease. In addition, our time-course experiments found a novel and significant upregulation of *DNMT3B* 48 hours after the addition of the maturation cocktail (mature day-2) (Figure 4 and Additional file 8A). Collectively, our data suggest *de novo* methylation upon the initiation of differentiation and demethylation upon the initiation of maturation, followed by *de novo* methylation during maturation.

Another mechanism for active demethylation is TET protein-mediated oxidation of 5mC to 5hmC, a potential intermediate for active DNA demethylation, followed by secondary reactions that eventually lead to restoration of cytosine. The expression of *TET1* was very low and there was no detectable change during the course of this experiment. However, the expression levels of *TET2* were highly upregulated as early as day 1 during monocyte to iDC differentiation (Figure 4B and Additional file 8B). The expression of *TET3* remained unchanged during the differentiation as well as maturation (Figure 4B). It has been previously shown that region-specific demethylation by TET protein through the production of 5hmC is promoted by PPARγ-induced PARylation in adipocytes [63]. Indeed, we found that many components within the PPARγ pathway, including PPARγ itself, were demethylated from monocyte to iDC (Figure 2A), suggesting that this pathway might also be involved in DC differentiation.

To further examine the temporal dynamics of DNA methylation changes, we used an ELISA-based methodology to examine global 5-mC and 5-hmC level dynamics during the course of monocyte differentiation and iDC maturation. We observed a trend of loss in gross levels of 5mC during the course of differentiation [see Additional file 9A, day-0 to day-4], consistent with what we have observed in purified cell populations. However, after the addition of maturation cocktail, there was a significant reduction in global 5mC (*P* = 0.044, mature day-1 versus day-4), followed by a trend of increase. This coincides with the upregulation of TETs and DNMTs, suggesting that TETs and DNMTs may be responsible for the dynamic changes in DNA methylation. However, the level of 5hmC was not significantly altered during DC differentiation although there was a trend of loss during maturation [see Additional file 9B, *P* = 0.052]. Parallel analysis of purified monocytes, iDCs and mDCs with this same approach also showed no significant changes in global 5hmC level [see Additional file 9C].

### Gene-specific demethylation dynamics regulate expression profiles

**Figure 5:**
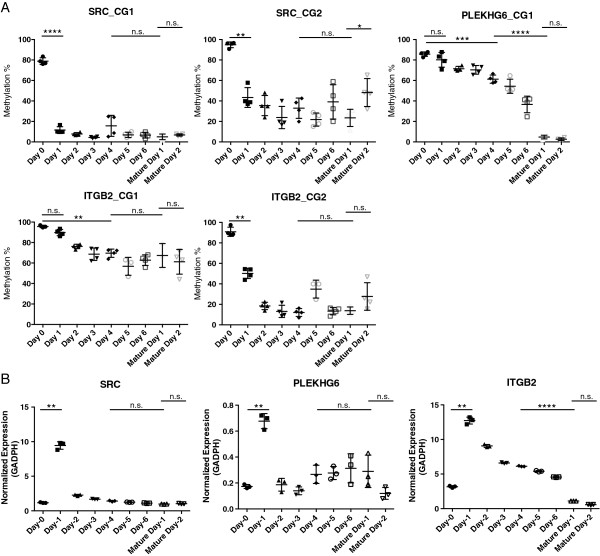
**Locus-specific dynamic DNA methylation and expression changes during dendritic cell (DC) differentiation and maturation.** DNA methylation at CG sites at the promoter of *SRC*, *PLEKHG6* and *ITGB2***(A)** and gene expression changes **(B)** were measured during the differentiation of monocyte into iDC (day 0 to day 4) and immature dendritic cell (iDC) maturation into mature dendritic cell (mDC) (day 4 to Mature day 1 to Mature day 2). iDCs without addition of maturation cocktail were grown for additional two days as well (day 5 and 6). Expression level in B was normalized to *GADPH*, and three biological replicates were used in each condition. One representative experiment among four replicates is shown here. Comparisons were made for Day 0 versus Day-1, Day-4 versus Mature Day-1, and Mature Day-1 versus Mature Day-2 using paired t-test. *P <0.05, **P <0.01, ***P <0.001, n.s., nonsignificant.

In order to better characterize the gene- and locus-specific early and late methylation modifications, we measured the methylation level at specific CG sites in *SRC*, *PLEKHG6* and *ITGB2*, which showed a huge erasure of DNA methylation (approximately 70% to 80%) either from monocytes to iDCs or from iDCs to mDCs during this time course. The two CG sites in *SRC* were demethylated as early as 24 hours after the addition of IL-4 and GM-CSF and stayed the same for rest of the experiment, suggesting that demethylation of this gene is an early active event in DC differentiation (Figure 5A). This demethylation was associated with significant upregulation of *SRC* expression at 24 hours (Figure 5B), consistent with previous reports (GSE7509, Figure 3B). This change in DNA methylation couples with an increase in the expression of *DNMT1*, *DNMT3A*, and *TET2* (Figure 4). In contrast, the CG site located at the 5′UTR of *PLEKHG6* was gradually demethylated with IL-4 and GM-CSF treatment, and was completely demethylated after the addition of the maturation cocktail, suggesting that the erasure was more of a passive, steady process occurring over a period of time. It is likely that the demethylation in *PLEKHG6* does not directly regulate gene expression, because, despite the differences in DNA methylation dynamics in *SRC* and *PLEKHG6*, their expression dynamics are similar (Figure 5A and B). The expression of *SRC* and *PLEKHG6* went down to basal levels after 48 hours and remained almost constant for the rest of the experiment, similar to previous reports (GEO: GSE7509) (Figure 5B). The methylation level of two CG sites in *ITGB2* (CG1 and CG2) was reduced to a stable level in 2 days (Figure 5A). However, the demethylation of CG1 is much slower than CG2, and the change of CG2 is more associated with the rapid regulation of *ITGB2* at 24 hours (Figure 5B). Consistent with the published dataset (GSE7509), the expression of *ITGB2* was significantly upregulated during the differentiation of monocytes into iDCs, and was significantly downregulated during their maturation. Collectively, our observations demonstrated that the alteration of DNA methylation for these CG sites does not occur at the same time, and their impacts on gene expression may differ. This suggests delicate, time-specific epigenetic programming during development and possibly site-specific mechanisms for DNA demethylation.

## Discussion

In order to enhance our understanding of DNA methylation in DC lineage commitment and cellular response to external stimuli, we established single nucleotide-resolution genome-wide and locus-specific DNA methylation studies for CD14^+^ monocytes, monocyte-derived dendritic cells (iDCs), and DCs matured by Jonuleit cocktail (mDCs). Dynamic DNA methylation changes, predominately an erasure of DNA methylation, occurred during these two *ex vivo* processes, particularly around enhancers and binding sites for transcription factors known for dendritic cell lineage specification. Besides the identification of previously studied genes and pathways involved in dendritic cell function, our analysis also revealed many novel candidate genes, which are likely important for dendritic cell differentiation and response to stimuli. A negative correlation between methylation changes at CG sites and the expression of nearby genes was found independent of the location of CG sites, suggesting interplay between these two regulatory mechanisms. Time-course studies of enzymes involved in DNA methylation maintenance revealed dynamic changes in the expression level of *TET2*, *DNMT1*, *DNMT3A* and *DNMT3B* during differentiation and maturation, indicating that these enzymes may account for such function-specific and location-specific variations in DNA methylation.

### Our analysis reveals novel candidates involved in dendritic cell (DC) differentiation and maturation

Dendritic cells are antigen-presenting cells that excel at activating naïve T-cells, and in their steady state they act as sentinels that detect pathogens. When activated by these pathogens, they initiate the body’s innate and adaptive immune responses through the secretion of cytokines along with the presentation of antigenic peptides on MHCs. The functions of the known genes detected in our analyses are consistent with the functional switch of DCs between the steady and activated states (Figure 2). In addition, we were able to uncover many novel candidate genes for studying DC differentiation and activation. One candidate is *PLEKHG6*, a gene that encodes a guanine nucleotide exchange factor that can activate small GTPase RHOG and RAC1, and form a complex with ezrin and MYH10. Ezrin recruits PLEKHG6 to the apical pole of epithelial cells, where PLEKHG6 induces the formation of microvilli and membrane ruffles [64]; this interaction between PLEKHG6 and ezrin is required for EGF-stimulated macropinocytosis. PLEKHG6 can also form a complex with MYH10 and RHOA, which is located at the cleavage furrow to advance furrow ingression during cytokinesis [65,66]. To date, no studies have addressed the role of PLEKHG6 in dendritic cell maturation. However, our observations of greatly reduced methylation levels at a CG site at the 5′-UTR of *PLEKHG6* as well as at CG sites in the 5′-UTR of its interacting partner *RHOG*, and the second intron of *MYH10* suggest that this complex may play a role in the acquisition of functions that are associated with a more mature DC phenotype [67,68]. Additionally, the methylation level at a CG site located at the 5′UTR of *ITGB2* (beta 2 integrin, which encodes integrin beta chain beta 2) was reduced by 74% (Figure 2A). The integrin beta chain beta 2 combines with the alpha L chain to form the integrin LFA-1, and combines with the alpha M chain to form the integrin Mac-1. LFA-1 play a central role in leukocyte (T cells) migration across blood vessel walls into lymph nodes and tissues, and it is a key participant in the immunological synapse [69,70]. However, the detailed role of LFA-1 in dendritic cell is not clear.

### TET2 and DNMTs may be responsible for the spatial and temporal DNA methylation changes

We observed that over 1,367 CG sites underwent demethylation from monocytes to iDCs, and 139 CG sites were demethylated during the transition from iDCs to mDCs. Detailed locus-specific DNA methylation measurement by pyrosequencing found that these methylation changes were locus- and time-specific (Figure 2 and Figure 5). Recently, it has been shown that *TET2* is highly expressed during monocyte differentiation and is required for active demethylation from monocytes to iDCs [71]. However, in this study, the expression of *TET2* (normalized to HPRT) remained nearly constant for 66 hours [71]. In contrast, we noticed a significant increase in the expression of *TET2*, as early as 24 hours after the introduction of IL-4 and GM-CSF. As the upregulation of *TET2* remained significant, even after normalizing with HPRT in our experiments [see Additional file 8D], this disparity could be due to the difference in the concentrations of IL-4 and GM-CSF used to generate iDCs. Further, siRNA-mediated knockdown of *TET2* in monocytes prevented active demethylation at specific loci during monocyte differentiation [71], which supports our observation that *TET2* expression is upregulated when demethylation occurs. In addition, a study conducted by Kallin *et al*. reported that TET2 binds to the promoter of *ITGB2* during myeloid transdifferentiation and knockdown of *TET2* was associated with an incomplete activation of *ITGB2 *[72], which is in line with our observation, supporting that *TET2* may influence *ITGB2* expression through promoter demethylation. In agreement with the previous reports, levels of *TET1* were undetectable, and furthermore, and no differences were observed in *TET3* expression levels, suggesting *TET2* as an important player in the erasure of DNA methylation during the differentiation of monocyte into iDCs.

Surprisingly, we observed significantly increased expression of *DNMT1* and *DNMT3A* upon the initiation of monocyte differentiation to iDCs, and significant downregulation of *DNMT1* upon maturation, followed by significant upregulation of *DNMT3B* during the maturation of iDCs to mDCs. DNMT1 is responsible for the maintenance of DNA methylation through mitotic divisions, while DNMT3A/3B are *de novo* methyltransferases that actively methylate DNA during multiple cellular processes [73-75]. It has been shown that knocking down *DNMT1* and *DNMT3B* or inhibition by azacytidine results in global demethylation [76]. Controversially, DNMTs were also proposed to function as deaminases and base excision enzymes to reduce DNA methylation [14,77]. Recently it has also been shown that DNMTs are capable of directly removing the hydroxymethyl moiety from 5-hmC and the methyl group from 5-mC *in vitro* or in a redox state-dependent manner [78,79]. Therefore, it is tempting to speculate that DNMTs have a dual role in regulating DNA methylation. They could actively demethylate DNA or *de novo* methylate DNA. Interestingly, the methylation level of a CG site located at the 5′ UTR of *DNMT3B* showed a gradual increase, suggesting that the expression of DNMT3B might also be regulated by DNA methylation (Figure 2A). Although inhibitors of DNMTs did not affect the active DNA methylation process during iDC formation [18], mDCs generated with DNMT inhibitors during iDC differentiation and maturation exhibited differences in surface marker expression and cytokine production [80], suggesting a possible regulatory role of DNMTs on some specific genes. Nevertheless, our data strongly support further investigation on the exact roles of DNMTs in DC differentiation and maturation, possibly on an individual gene basis at a specific timing.

### Clinical implications and future directions

Our studies suggest that DNA methylation regulates key genes and pathways in dendritic cell differentiation and maturation. The feasibility of large-scale *ex vivo* generation of DCs from patients’ monocytes allows for therapeutic application of *ex vivo*-cultured DCs to bypass the dysfunction of endogenous DCs, restore immune surveillance, induce cancer regression and stabilization, and delay or prevent its recurrence. While the most common paradigm of the therapeutic application of DCs reflects their use as cancer ‘vaccines’, additional and potentially more effective possibilities include the use of patients’ autologous DCs as part of more comprehensive therapies. This involves *in vivo* or *ex vivo* induction of tumor-reactive T cells, which would counteract systemic and local immunosuppression in tumor-bearing hosts. Given the reversibility of DNA methylation by demethylating reagents and the possibility of gene-specific DNA manipulation using long non-coding RNA [81], our observations suggest a novel approach to regulate this process. *Ex vivo-*cultured DCs can therefore be instructed to acquire distinct and optimal functions relevant to the induction of effective cancer immunity (DC polarization), such as the induction of different effector functions or different homing properties of tumor-specific T cells. Our data also support a possible role for TET enzymes and DNMTs in DC differentiation and maturation, suggesting that both could be targeted to modify the treatment efficacy of DC vaccines.

## Conclusions

Locus- and time-specific DNA demethylation occurs during the *ex vivo* differentiation of human monocytes into immature dendritic cells and their maturation by an FDA-approved cytokine cocktail. Many of such DNA methylation changes happen near promoters, enhancers and transcriptional factor binding sites, and are correlated with an increase in gene expression of nearby genes. Importantly, the expression level of *TET2*, *DNMT1*, *DNMT3A* and *DNMT3B* are upregulated in a time-dependent manner, which happens concurrently with the DNA methylation changes, suggesting that these enzymes may be responsible for the precise spatial and temporal regulation of DNA methylation levels.

## Methods

### *Ex vivo* generation and maturation of dendritic cells

This study was approved by Cincinnati Children’s Hospital Medical Center (CCHMC) Institutional Review Board and characterized as not human subject research (CCHMC, IRB #2012-0136). Peripheral Blood Mononuclear Cells (PBMCs) were isolated from the blood samples of anonymous healthy donors from the Hoxworth Blood center at University of Cincinnati using Ficoll-Paque Plus density gradient centrifugation. Monocytes were then isolated from PBMCs by CD14^+^ sorting with Miltenyi beads (Miltenyi Biotec Inc., San Diego, CA, USA) and cultured in the presence of GM-CSF (1000U/ml) and IL-4 (500U/ml) for 4 days to induce differentiation into mostly immature monocyte-derived DCs (iDCs) [82-85]. These DCs were matured by culturing for an additional two days in the presence of a Jonuleit cocktail (IL1β, IL1α, IL6, TNFα and PGE2) [5]. Harvested cells were sorted to obtain pure populations of the desired cell, before being subjected to DNA methylation analysis using microarrays and pyrosequencing. Cells used in the time-course experiment were directly subjected to DNA/RNA extraction.

### Flow cytometry

Immature DCs and mature DCs were stained with a LIVE/DEAD Fixable Dead Cell Stain Kit (Life Technologies, Grand Island, NY, USA), then fixed with 2% PFA. After fixation, the cells and appropriate controls were prepared. All samples were stained with HLA-DR-PE/Cy7 (Biolegend, San Diego, CA, USA), CD83-AF647 (Biolegend, San Diego, CA, USA), and CD83-PE (Biolegend, San Diego, CA, USA). Cells were analyzed on a BD LSR II (BD Biosciences, San Jose, CA, USA). Flow data were analyzed using FlowJo v9.6.1 software.

### Cell sorting

CD14^+^ cells presorted by CD14 MicroBead Kit (Miltenyi Biotec Inc., San Diego, CA, USA), immature DCs, and mature DCs were stained with CD14-FITC (Biolegend, San Diego, CA, USA), HLA-DR-PE/Cy7 (Biolegend, San Diego, CA, USA), CD83-AF647 (Biolegend, San Diego, CA, USA) and Live/Dead-7-AAD (eBioscience, San Diego, CA) and sorted on a MoFlo XDP (Beckman Coulter Inc., Brea, CA, USA). Cells were collected in 100% FBS and pelleted for DNA/RNA extraction.

### DNA and RNA isolation

DNA and RNA were isolated from the same samples using AllPrep DNA/RNA Micro kit (Qiagen, Valencia, CA, USA) according to the manufacturer’s protocols.

### Illumina Infinium 450 K beadchip DNA methylation analysis

Genomic DNA was bisulfite treated and assayed by the Illumina Infinium HumanMethylation450 BeadChip (Illumina, San Diego, CA, USA) at the Genomics and Microarray lab at University of Cincinnati Medical Center. Quality of the arrays was assessed using sample-independent and dependent internal control probes included on the array for staining, extension, hybridization, specificity and bisulfite conversion. One immature DC sample exhibited low intensity for all controls, suggesting a problematic quality of this array. Therefore, this sample was excluded from subsequent analyses. The remaining 11 samples all had >98% CG sites detected at *P* = 0.01 level, and >80% bisulfite conversion rate. The signal intensities were background-adjusted, normalized, and used to calculate beta values using the methylation module. The following CG sites were excluded from analysis: 1) CG sites that were not detected in all samples at *P* = 0.01 level; 2) CG sites on X and Y chromosomes; and 3) CG sites assayed by less than five beads in one or more samples. These procedures resulted in 11 samples and 343,023 CG sites for analyses.

For each of the CG sites, the beta values were compared between monocytes and immature DC, and between immature DC and mature DC using paired t tests. Given the small sample size of the methylation array and the importance of the actual methylation level, we used both *P* values and absolute differences in beta values to determine DMPs. A DMP was defined if *P* value ≤0.05 and absolute differences in beta values ≥0.1. All microarray data have been deposited to NCBI Gene Expression Omnibus (GSE59796).

To better understand the biological meaning behind the methylome changes, the list of DMPs was imported into Ingenuity Pathway Analysis (Ingenuity Systems, Redwood City, CA) for pathway mapping, gene network detection, and upstream regulator identification. A cutoff of 0.05 was used for statistical significance in IPA analysis.

### Bisulfite pyrosequencing

A total of 200 ng of genomic DNA from each sample was treated with bisulfite using an EZ DNA methylation-Gold Kit (ZYMO research, Irvine, CA, USA) according to the manufacturer’s specifications. The bisulfite-treated genomic DNA was amplified by PCR using unbiased nested primers and DNA methylation was measured by quantitative pyrosequencing using a PyroMark Q96 MD (Qiagen, Valencia, CA, USA). The DNA methylation percentage at each CG site was determined using the Pyromark CpG methylation software (Qiagen, Valencia, CA, USA). *Sss*I-treated human genomic DNA was used as 100% methylation control and human genomic DNA amplified by GenomePlex Complete Whole Genome Amplification (WGA) Kit (Sigma, St. Louis, MO, USA) was used as the nonmethylated DNA control. Primer sequences used for the bisulfite pyrosequencing reactions are shown in Additional file 10, as well as the chromosomal coordinates in the University of California at Santa Cruz February 2009 human genome assembly for each CG site measured. The annealing temperature used for all PCR reactions was between 50°C and 55°C.

### Affymetrix microarray expression analysis

Microarray gene expression profile with Affymetrix hgu133plus2 has been preprocessed by RMA (Robust Multi-array Average) procedure, an algorithm tool to convert the raw probe level data into a normalized expression matrix containing annotated genes [86]. Three groups (immature DC (#3), mature DC (#4), and monocyte (#3)) of our major interest to be compared have biological replicates. To obtain normalized expression measurement, raw intensity values on all corresponding arrays with CEL files were adjusted by background correction, log2-transformation, normalization across arrays under the rationale of quantile normalization that all distributions are the same, and summarization on single gene-level intensity from combined intensity values in the probe set by making use of median polishing. All pairwise comparisons to test two groups were carried out through a t-test [87] procedure in the genefilter R package, and a simultaneous multi-group comparison was performed by LIMMA [88]. All analyses were on the basis of Bioconductor and R packages [86].

### Association of DNA methylation with gene expression

Microarray data generated by different probes of one gene were averaged and used as the expression at gene-level. The gene-level expression was compared between monocytes and immature DC using paired t tests, and between immature and mature DCs using two-sample t tests. False-discovery rate (FDR) was calculated with the q value package. Differential expression was considered when q values ≤ 0.05. The changes in expression levels of differentially expressed genes were then correlated with changes in beta values of DMPs, and Pearson’s correlation coefficients were reported for CG sites in the island, shore, shelf, and open sea.

### Quantitative PCR

RNA was isolated from the Qiagen Allprep kit mentioned above, according to manufacturer’s instructions. cDNA was synthesized using the Superscript III kit (Life Technologies, Grand Island, NY, USA) using random hexamers. Amplifications were performed using SYBR Green PCR core reagents (Life Technologies, Grand Island, NY, USA), and transcript levels were quantified using an ABI 7900 Sequence Detection Systems (Life Technologies, Grand Island, NY, USA). Mean Ct value of triplicate reaction was normalized against mean Ct value of *GAPDH* and/or *HPRT*. Primer sequences are included in Additional file 11.

### Identification of enriched transcription factor binding motifs

We used a custom ‘library’ of 1,907 human transcription factor (TF) binding motifs to identify TF motifs enriched in a ‘positive’ set of sequences (those with DNA methylation changes), compared to a ‘negative’ set (a matched set of sequences without DNA methylation changes). The library consists of motifs taken from databases such as Transfac [89], JASPAR [90], UniPROBE [91], and FactorBook [92], as well as motifs collected from individual studies such as Jolma *et al. *[93] and Weirauch *et al. *[94]. The manuscript describing this motif library is currently in review. We used a 51 base window (25 bases on either side of DMP) to construct all sequence sets. We then estimated the enrichment of each motif using the Pscan [95], which ranks all motifs by *P* value, based on the average best score of the motif in the sequences of the positive set, compared to the negative set. About 2,000 motifs were tested, so a cutoff of *P* <0.000005 was used (corresponding to a *P* value of 0.01, corrected using Bonferroni’s conservative method). Nearly identical results were achieved using the HOMER algorithm [96] (data not shown). For example, the top 13 motifs for the monocyte to iDC transition are all AP-1 related (for example, FOS, JUN), all with HOMER *P* values <10^-25^. Likewise, the top 23 enriched motifs for the iDC to mDC transition all contain a GGAA core (for example, BCL11A, SPI, and RELA).

## Abbreviations

cDC: conventional dendritic cell; DC: dendritic cell; DMP: differentially methylated points; DNMT: DNA-methyltransferase; ELISA: enzyme-linked immunosorbent assay; FACS: fluorescence-activated cell sorting; FDR: false discovery rate; GM-CSF: granulocyte/macrophage colony-stimulating factor; HPRT: hypoxanthine-guanine phosphoribosyltransferase; iDC: immature dendritic cell; IL-4: interleukin 4; LPS: lipopolysaccharide; mDC: mature dendritic cell; TET: ten-eleven translocation methylcytosine dioxygenase; TF: transcription factor; 5-mC: 5-methylcytosine; 5-hmC: 5-hydroxymethylcytosine; IPA: Ingenuity Pathway Analysis; PBMC: Peripheral Blood Mononuclear Cell.

## Competing interests

The authors declare that they have no competing interests.

## Authors’ contributions

XZ performed genome-scale microarray processing and statistical analysis; AU performed cell-sorting and extracted DNA/RNA; HKS performed gene-specific DNA methylation, gene expression analysis, and global measurement of 5mC and 5hmC; MAL assisted in cell sorting; SO analyzed gene expression microarray datasets; HXZ participated in analyzing gene expression microarray datasets; XC performed transcription factor binding motif analysis; MTW participated in transcription factor binding motif analysis; EMJ participated in the design of the experiments; HJ conceived and designed the experiments and wrote the paper with the assistance of all other authors. All authors read and approved the final manuscript.

## Supplementary Material

Additional file 1 **Prospective isolation of monocyte, immature dendritic cells (iDCs) and mature dendritic cells (mDCs) using fluorescence activated cell sorting (FACS).** (A) Schematic showing the protocol used to generate iDCs and mDCs *ex vivo* from monocytes. (B) Cell populations were purified based on the combination of cell-surface marker expressions defined as follows: monocyte, CD14^+^; iDCs, HLA-DR CD83^-^; mDCs, HLA-DR^high^ CD83^+^. Click here for file

Additional file 2 **Differentially methylated points (DMPs) identified comparing monocyte to immature dendritic cell (iDC), and comparing iDC to mature dendritic cell (mDC).** (A) monocyte > iDC, with the same direction of change in associated genes. (B) monocye < iDC, with the same direction of change in associated genes. (C) monocyte versus iDC, with mixed directions of change in associated genes. (D) iDC > mDC, with the same direction of change in associated genes. (E) iDC < mDC, with the same direction of change in associated genes. Click here for file

Additional file 3 **Significantly enriched transcription factor (TF) binding sites around differentially methylated points (DMPs).** (A) monocyte versus immature dendritic cell (iDC) DMPs. (B) iDC versus mature dendritic cell (mDC) DMPs. Click here for file

Additional file 4 **Ingenuity pathway analyses to identify significantly enriched gene sets and pathways with DNA methylation changes during DC differentiation from monocyte.** (A) monocyte > immature dendritic cell (iDC), with the same direction of change in associated genes. (B) monocye < iDC, with the same direction of change in associated genes. (C) monocyte versus iDC, with mixed directions of change in associated genes. Click here for file

Additional file 5 **Ingenuity pathway analyses to identify significantly enriched gene sets and pathways with DNA methylation changes during dendritic cell (DC) maturation.** (A) immature dendritic cell (iDC) > mature dendritic cell (mDC), with the same direction of change in associated genes. (B) iDC < mDC, with the same direction of change in associated genes. Click here for file

Additional file 6 **Ingenuity pathway analyses to identify upstream regulator of genes with DNA methylation changes.** (A) monocyte versus immature dendritic cell (iDC). (B) iDC versus mature dendritic cell (mDC). Click here for file

Additional file 7 **Correlation of DNA methylation changes with gene expression alterations during dendritic cell (DC) maturation.** The changes in expression levels of differentially expressed genes were correlated with changes in beta values of differentially methylated points (DMPs), and Pearson’s correlation coefficients were reported for CG sites in the island shore, shelf and open sea, as specified in the figure. When all CG sites combined, n = 46, Pearson r = -0.26, *P* = 0.0863. Click here for file

Additional file 8 **Dynamic changes in expression levels of ten-eleven translocation methylcytosine dioxygenase 2/3 (TET2/3) and DNA-methyltransferases (DNMTs).** Gene expression levels of *DNMT1*, *DNMT3A*, and *DNMT3B* (A and C), *TET2* and *TET3* (B and D) of one donor among four donors were plotted. Expression level was normalized to *GADPH* in A and B, to *HPRT* in C and D. Three technical replicates were used in each condition. Comparisons were made for Day 0 versus Day-1, Day-4 versus Mature Day-1, and Mature Day-1 versus Mature Day-2 using paired t test. *P <0.05, **P <0.01, ***P <0.001, ****P <0.0001. Click here for file

Additional file 9 **Global changes in 5mC and 5hmC during monocyte differentiation into immature dendritic cells (iDCs) and iDC maturation into mature dendritic cells (mDCs).** A) 5-mC and B) 5-hmC were measured by ELISA as described in Materials and Methods in the same time course experiment as in Figure 5. C) 5-hmC in fluorescence-activated cell sorting (FACS) purified monocytes, iDCs and mDCs. Paired student t test was used to compare all the different groups. Results are shown as mean ± SD. Click here for file

Additional file 10 Primers for bisulfite pyrosequencing and the CpG sites interrogated. Click here for file

Additional file 11 Primers for qPCR. Click here for file
