# Supplementary figures and images for: DNA methylation dynamics during ex vivo differentiation and maturation of human dendritic cells
Source: Epigenetics Chromatin. 2014 Aug 20;7:21. doi: 10.1186/1756-8935-7-21 (PMC4144987; doi:10.1186/1756-8935-7-21)

A.

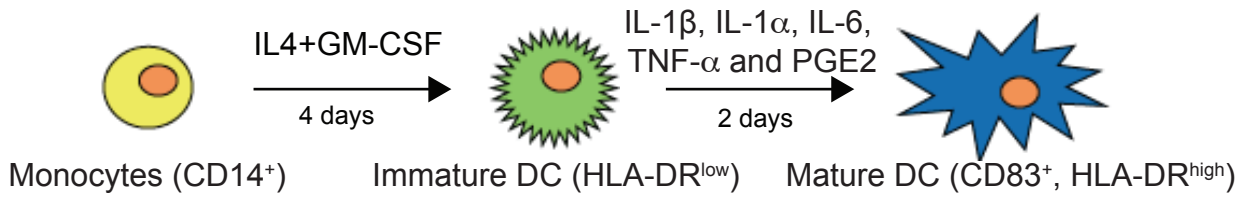

B.

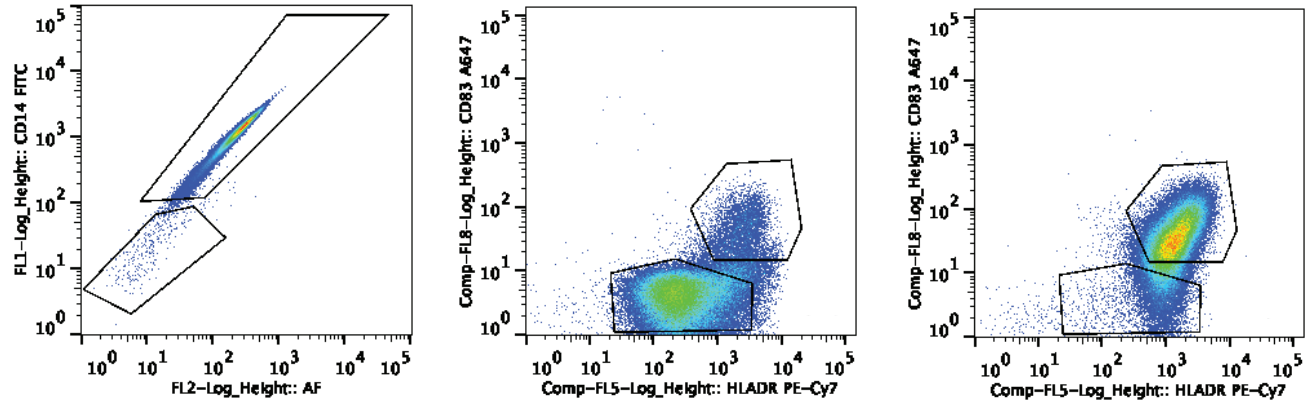

Supplemental figure 1

Supplement: Additional file 1 — Prospective isolation of monocyte, immature dendritic cells (iDCs) and mature dendritic cells (mDCs) using fluorescence activated cell sorting (FACS). (A) Schematic showing the protocol used to generate iDCs and mDCs ex vivo from monocytes. (B) Cell populations were purified based on the combination of cell-surface marker expressions defined as follows: monocyte, CD14+; iDCs, HLA-DR CD83-; mDCs, HLA-DRhigh CD83+. [file 1756-8935-7-21-S1.pdf]

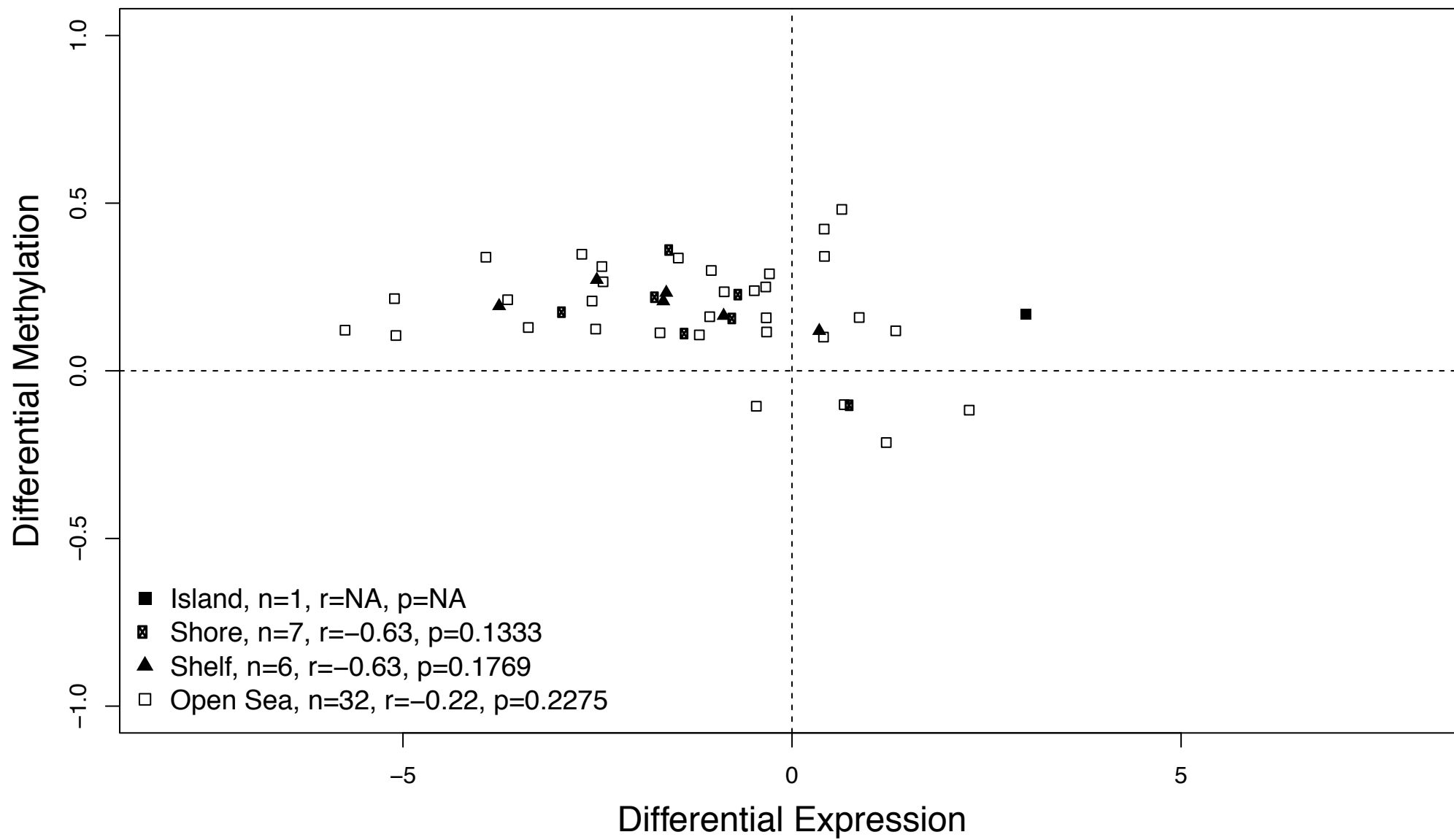

Supplement: Additional file 7 — Correlation of DNA methylation changes with gene expression alterations during dendritic cell (DC) maturation. The changes in expression levels of differentially expressed genes were correlated with changes in beta values of differentially methylated points (DMPs), and Pearson’s correlation coefficients were reported for CG sites in the island shore, shelf and open sea, as specified in the figure. When all CG sites combined, n = 46, Pearson r = -0.26, P = 0.0863. [file 1756-8935-7-21-S7.pdf]

A.

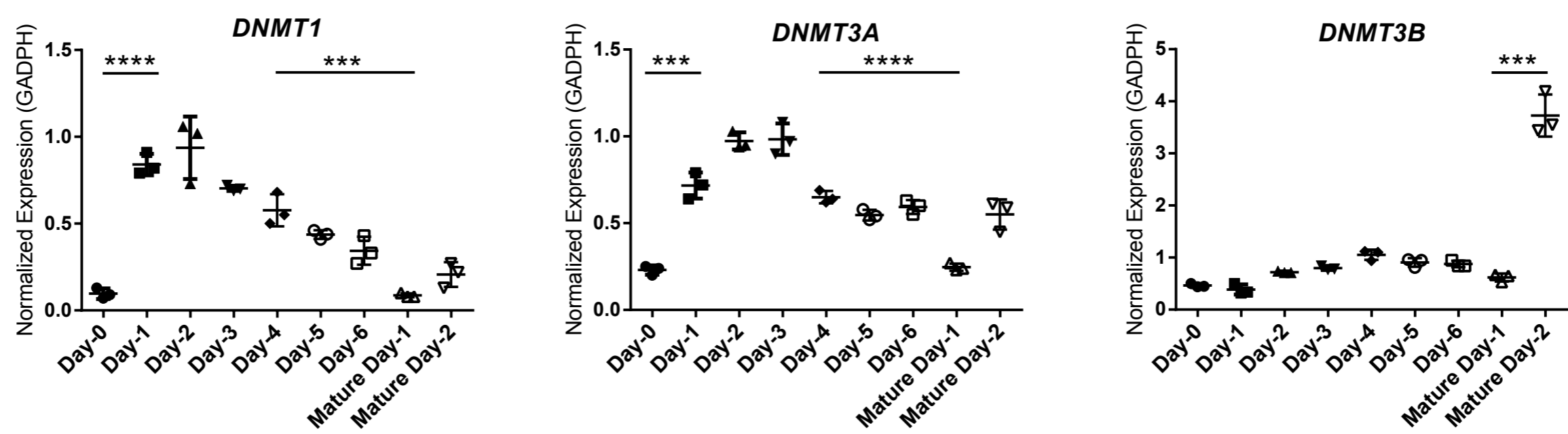

B.

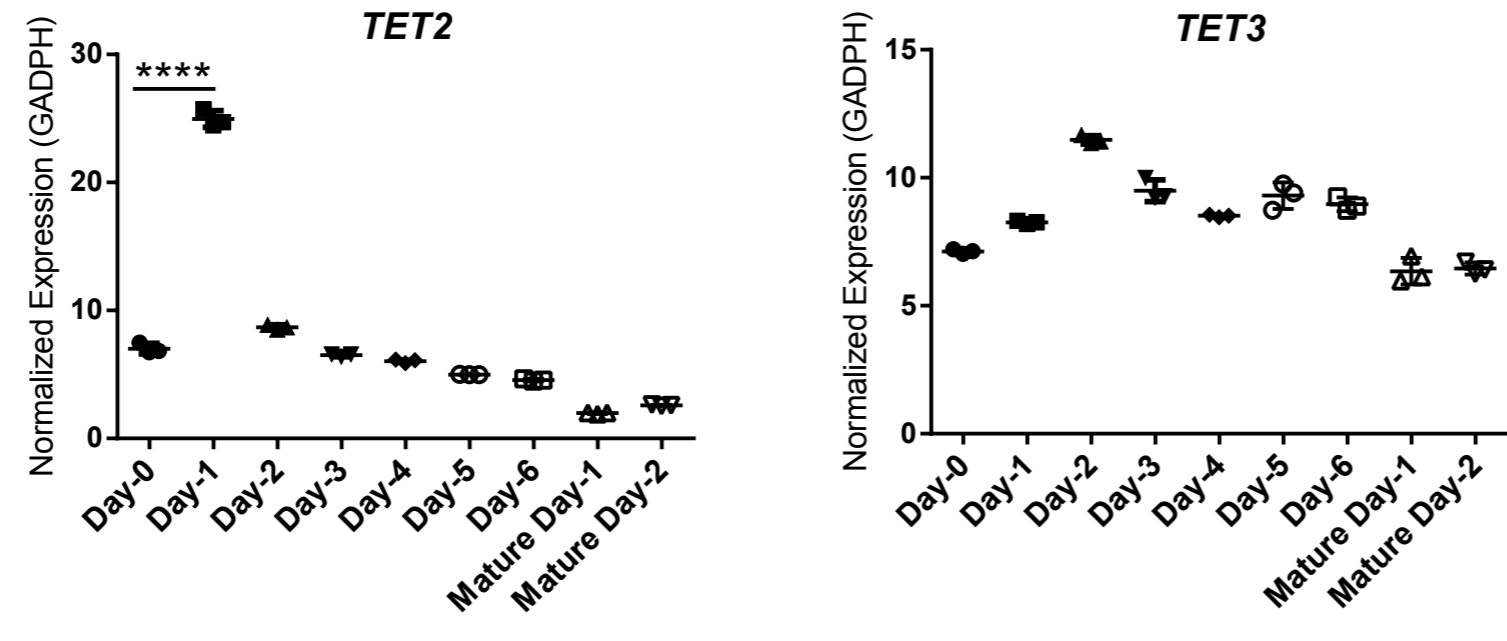

C.

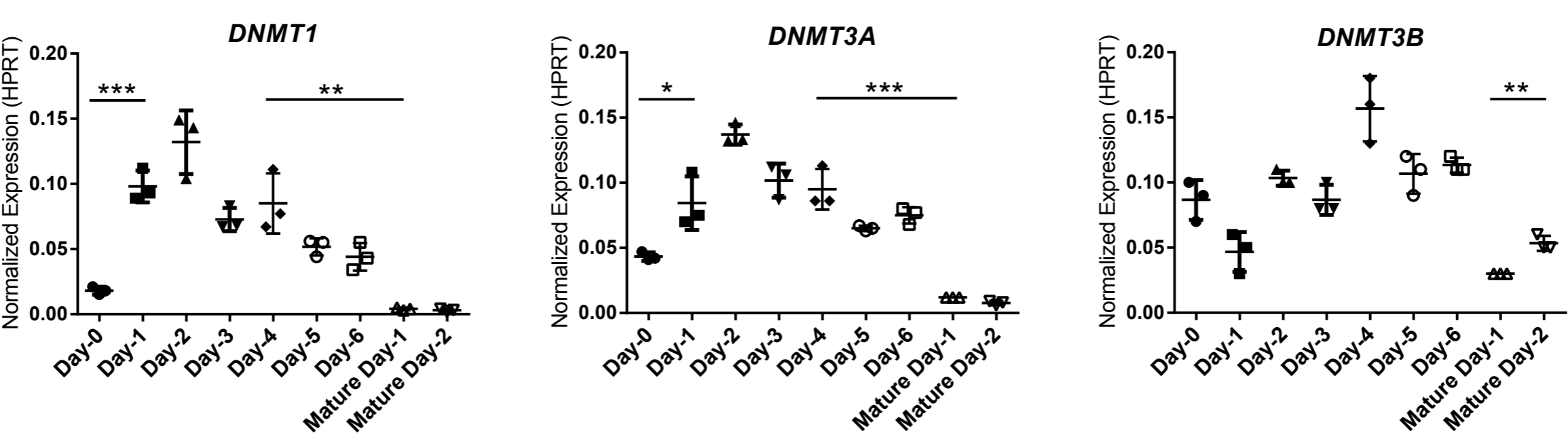

D.

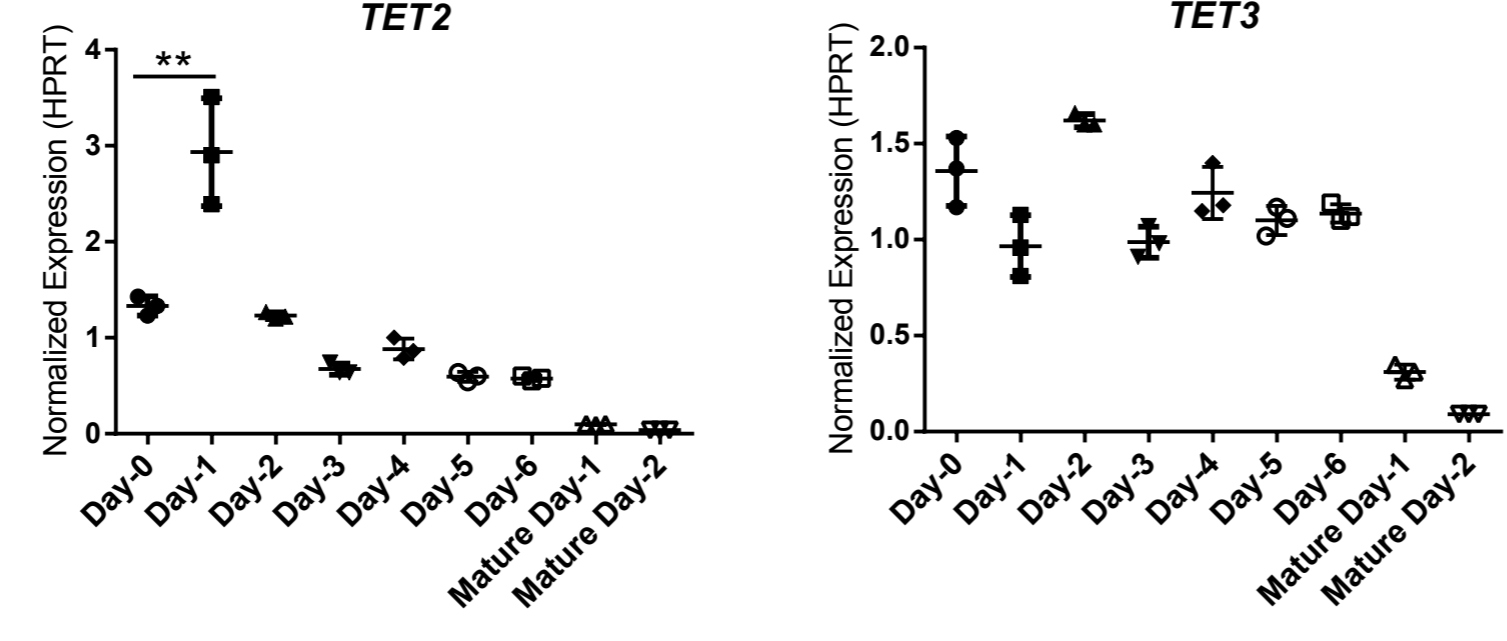

Supplement: Additional file 8 — Dynamic changes in expression levels of ten-eleven translocation methylcytosine dioxygenase 2/3 (TET2/3) and DNA-methyltransferases (DNMTs). Gene expression levels of DNMT1, DNMT3A, and DNMT3B (A and C), TET2 and TET3 (B and D) of one donor among four donors were plotted. Expression level was normalized to GADPH in A and B, to HPRT in C and D. Three technical replicates were used in each condition. Comparisons were made for Day 0 versus Day-1, Day-4 versus Mature Day-1, and Mature Day-1 versus Mature Day-2 using paired t test. *P <0.05, **P <0.01, ***P <0.001, ****P <0.0001. [file 1756-8935-7-21-S8.pdf]

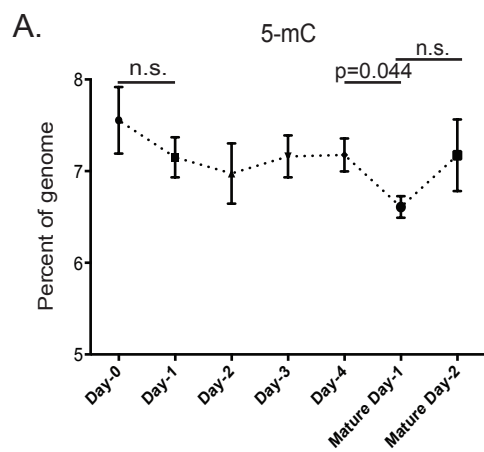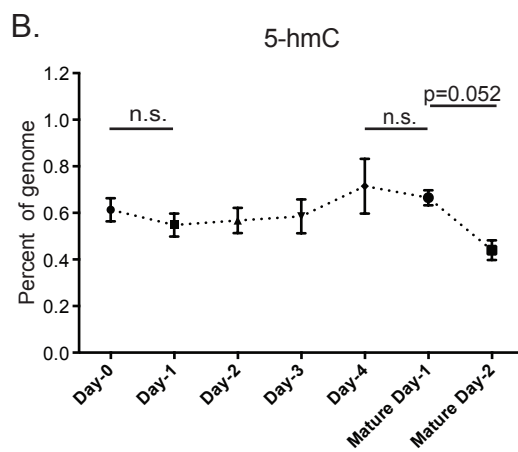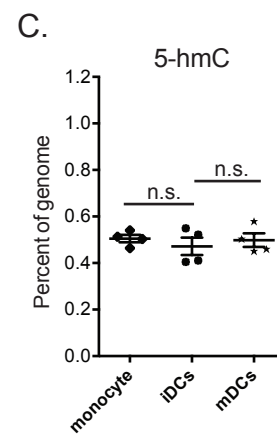

Supplement: Additional file 9 — Global changes in 5mC and 5hmC during monocyte differentiation into immature dendritic cells (iDCs) and iDC maturation into mature dendritic cells (mDCs). A) 5-mC and B) 5-hmC were measured by ELISA as described in Materials and Methods in the same time course experiment as in Figure 5. C) 5-hmC in fluorescence-activated cell sorting (FACS) purified monocytes, iDCs and mDCs. Paired student t test was used to compare all the different groups. Results are shown as mean ± SD. [file 1756-8935-7-21-S9.pdf]
